# A Technique for Difficult Intraoperative Dislocation of Hip Prosthesis in Revision Total Hip Arthroplasty

**DOI:** 10.7759/cureus.65600

**Published:** 2024-07-28

**Authors:** Philip K Johnson, Justin Lapow, Andrew R Grant, Peter Lementowski

**Affiliations:** 1 Orthopedic Surgery, New York Medical College, Valhalla, USA; 2 Orthopedic Surgery, Westchester Medical Center, Valhalla, USA; 3 Orthopedic Surgery, University of Wisconsin, Madison, USA; 4 Orthopedic Surgery, New England Baptist Hospital, Boston, USA

**Keywords:** surgical hip dislocation, challenging surgical dislocation, two-stage revision arthroplasty, intraoperative hip dislocation, revision total hip arthroplasty

## Abstract

There are instances where a patient’s prosthetic hip is unable to be dislocated intraoperatively during a conversion or revision arthroplasty, despite scar removal and standard dislocation maneuvers. We describe a technique that involves an in situ disassociation of the femoral head component from the trunnion without the need for additional osteotomies. This maneuver may be beneficial in cases of protrusio, muscular stiffness, high soft tissue tension, arthrofibrosis, and ankylosis due to heterotopic ossification, as well as cases that involve a large femoral head or acetabular constraint. We also present a case of a 61-year-old male with a chronic prosthetic hip infection who underwent a two-stage revision surgery where this technique was utilized.

## Introduction

Several challenges may occur during adequate surgical exposure and hardware removal during the revision total hip arthroplasty (rTHA) surgery, such as difficulty with the dislocation of the prosthetic head from the acetabular cup [1-3]. This is often due to a combination of factors such as large body habitus, high soft tissue tension, or excessive constraint from components designed to reduce clinical dislocation (i.e., large head, dual mobility liners, or constrained liners) [4,5]. In such cases of difficult hip dislocation, excessive torsional forces significantly increase the risk for periprosthetic fracture [6,7]. The incidence of intraoperative femur fracture has previously been reported to be 0.1-5.4% during primary THA and 3.6-45.9% during rTHA [8]. Thus, techniques for managing intraoperative hip dislocations during revision surgery may help reduce the risk of intraoperative periprosthetic fracture and therefore improve patient outcomes.

Previous literature has described the use of trochanteric osteotomies to facilitate surgical exposure and dislocation of the hip during rTHA surgery [9-11]. In general, the trochanteric osteotomy can be divided into three categories: the standard trochanteric osteotomy, the trochanteric slide osteotomy, and the extended trochanteric osteotomy [9]. Archibeck et al. reported that both the standard trochanteric osteotomy and trochanteric slide may be useful techniques for achieving dislocation in rTHA cases with acetabular component protrusio, while the extended trochanteric osteotomy may be useful in the attainment of dislocation of a stiff hip [9]. Sierra and Cabanela described the use of trochanteric osteotomy along with the removal of a thin rim of peripheral acetabular bone to allow for hip dislocation in the setting of extreme prosthetic protrusio during the conversion of hemiarthroplasty to THA [11]. Furthermore, Miner et al. reported the use of an extended trochanteric osteotomy to aid in dislocation of the hip during rTHA when extensive soft tissue scarring or heterotopic calcification was present [10]. Although the trochanteric osteotomy has been shown to be an effective technique for facilitating intraoperative dislocation of the hip, it has been associated with complications, including intraoperative or postoperative femur fracture, fragment nonunion, trochanteric bursitis, heterotopic ossification, and recurrent hip dislocation [9,10,12].

In cases where intraoperative dislocation becomes a challenge, we have utilized a technique that allows for sequential dislocation and retrieval of the components without the need for osteotomy. To the best of our knowledge, this is yet to be described in the literature.

## Case presentation

**Figure 1:**
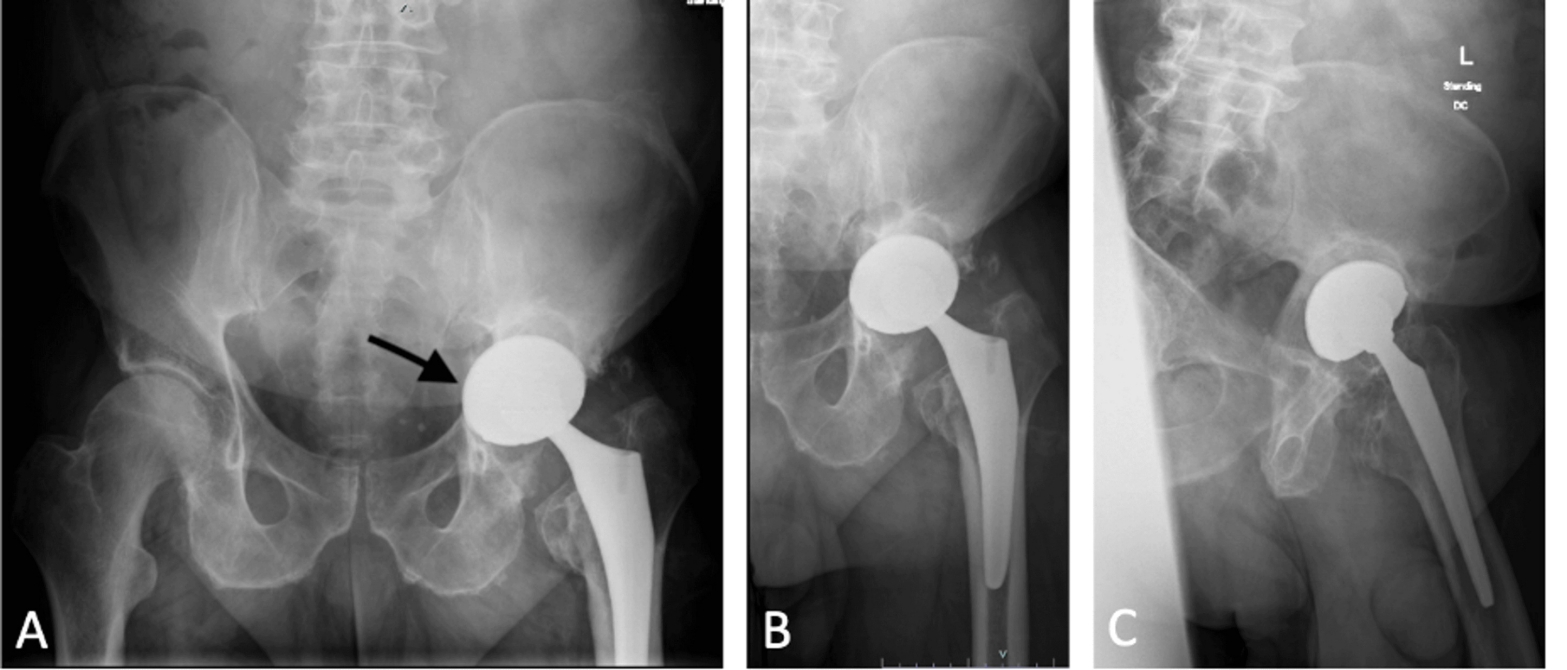
Pelvis X-rays (A) AP pelvis; (B) AP left hip; (C) lateral left hip radiographs reveal a press-fit THA with acetabular protrusio (black arrow) and medial wall loss.

The patient was informed that deidentified case data would be submitted for publication. Consent was appropriately obtained. The patient is a 61-year-old male community ambulator who underwent a left total hip arthroplasty five years prior at an outside institution. The patient was referred to our center due to a three-year history of groin pain, progressive difficulty bearing weight, leg shortening, and an acute “mechanical block” that happened several months prior. Initial pelvic and hip radiographs revealed a non-cemented THA with medial protrusion of the acetabular component into the pelvis (Figure 1).

**Figure 2:**
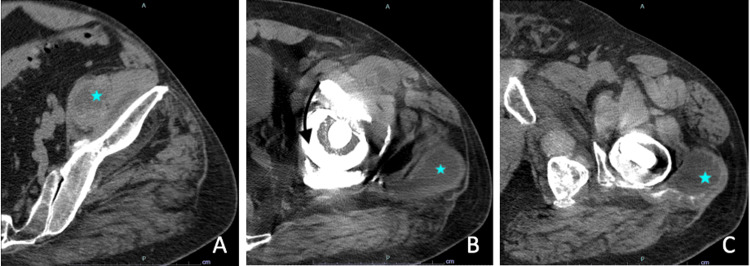
Successive axial cuts of CT scan left hip without contrast (A) Psoas fluid collection (blue star); (B) acetabular protrusio and medial wall loss (black arrow), along with soft tissue fluid collections around the iliopsoas and a large collection around the greater trochanter to the level of the fascia (blue star); (C) fluid collection around the greater trochanter (blue star).

A left hip CT scan showed additional soft tissue fluid collections around the iliopsoas and about the greater trochanter, indicative of a possible pseudotumor (Figure 2).

Initial laboratory studies obtained included a white blood count (WBC) of 5.8 k/mm^3^, an erythrocyte sedimentation rate (ESR) of 5 mm/h, and a C-reactive protein (CRP) of 0.5 mg/dL. At a subsequent follow-up visit, an in-office aspiration was performed, which yielded 20 mL of clear serosanguinous fluid without signs of purulence or metallosis was obtained due to the ballotable nature of his effusion as well as a pressure sensation. The patient experienced immediate relief from his pain. Despite a negative cell count on the aspiration, his cultures eventually grew *Cutibacterium acnes*. The patient was, therefore, scheduled for a stage I explantation and the placement of an articulating antibiotic cement spacer.

**Figure 3:**
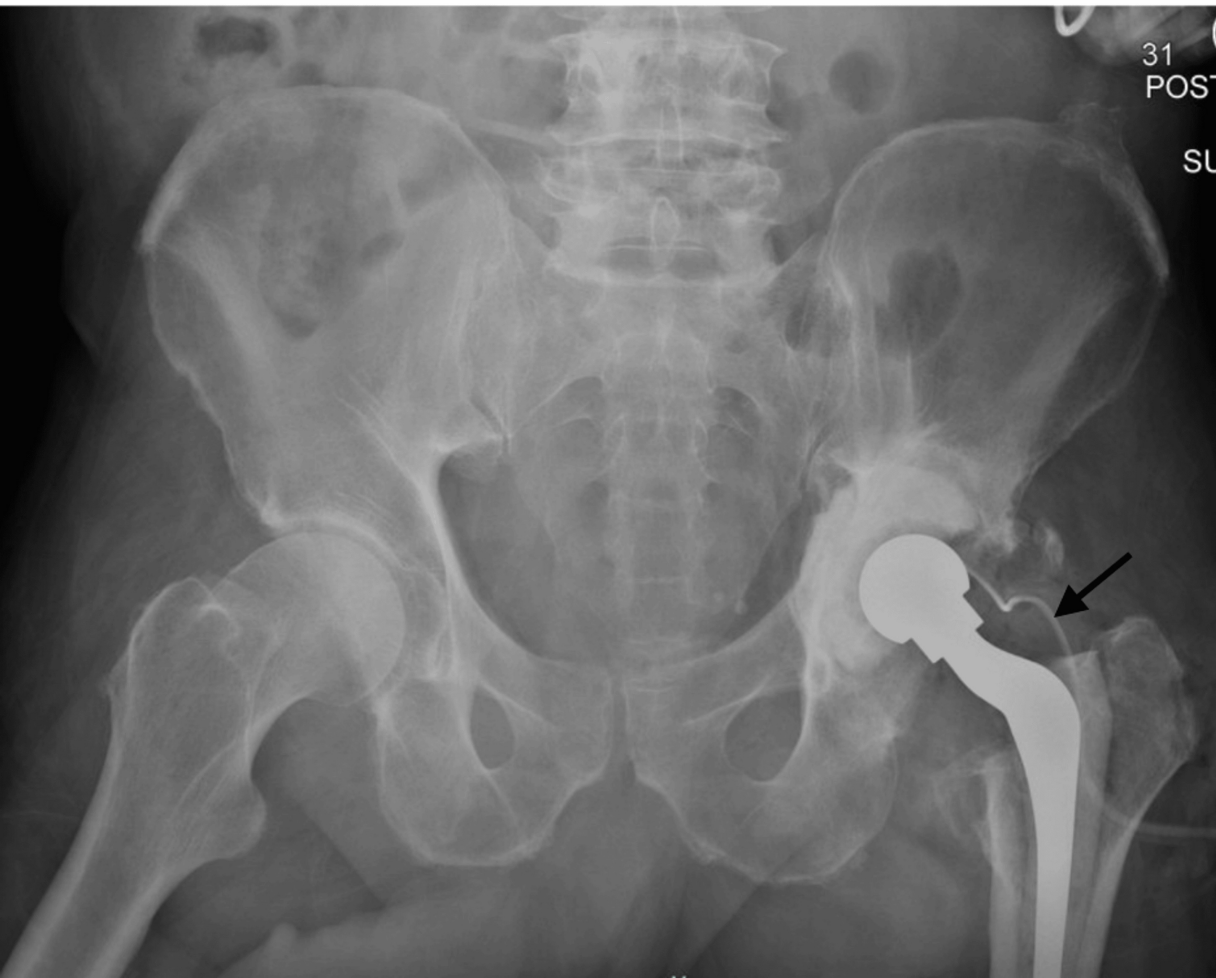
Postop-operative AP pelvis radiograph Demonstrates a DePuy Prostalac cemented polyethylene cup, size 4 + 13 mm offset Prostalac stem and size 32 mm Articul/eze™ metal head, and visible drain.

During surgery, a large cystic mass about the greater trochanter was excised, and a large caseating mass with significant fibrosis and nonviable tissue about the proximal femur and acetabulum was debrided. A dual mobility component with significant trunnionosis and a grossly loose acetabular component were found. Once all components were removed and thorough irrigation and debridement were performed, an articulating antibiotic spacer was implanted (Figure 3). The patient was then treated with IV antibiotics through a peripherally inserted central catheter line.

**Figure 4:**
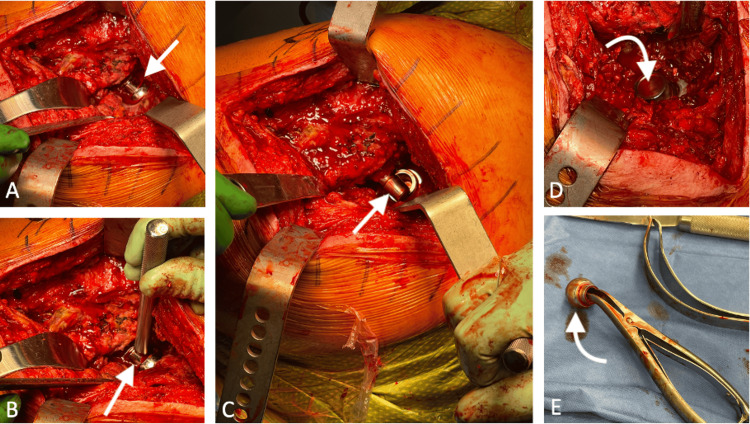
Intraoperative photographs (A) Initial femoral head component within constrained polyethylene liner (white arrow); (B) bone tamp placed on femoral head/neck junction (white arrow); (C) disassociation of trunnion (white arrow) from femoral head; (D) femoral head (white arrow) still intact in the polyethylene; (E) removal of femoral head (white arrow) using a small lamina spreader.

He returned to the OR several months later for his stage II left hip revision and the removal of an antibiotic cement spacer. The prosthetic femoral head spacer was unable to be dislocated despite scar tissue removal, further capsular releases, dislocation maneuvers, and the use of a bone hook. This was somewhat anticipated due to the cementation of the semi-constrained polyethylene cup in a medial position in the area of the previous defect, as well as improved soft tissue tension. We then proceeded to perform the in-situ femoral head disassociation technique. With the leg in a neutral position, a bone tamp was used to provide an axial force to the femoral head at its junction with the trunnion. This allowed for the dissociation of the morse taper. A bone hook was then placed about the shoulder of the femoral spacer, and in-line traction caused the femoral neck to be dislodged from the head, which remained in the semi-constrained polyethylene cup. The femoral head was then easily retrieved with the use of a small lamina spreader (Figure 4). Once this was complete, the leg was now able to be freely moved without fear of creating a distal femoral peri-prosthetic fracture. The remaining aspects of the second stage of surgery were then performed, consisting of antibiotic spacer removal, debridement, and placement of revision components.

## Discussion

We describe a technique in which a bone tamp is utilized to dissociate the morse taper between the prosthetic femoral head and the trunnion prior to hip dislocation, thus leaving the femoral head in situ within the acetabular cup. Dislocation of the femoral component can then be achieved with axial traction on the leg and the use of a bone hook about the shoulder of the prosthesis to provide direct traction. Once the junction is almost cleared, additional flexion and internal rotation will dislocate the trunnion out of the head posteriorly. The femoral head can then easily be removed with an instrument such as a small laminar spreader. This process eliminates the need for excessive torsional forces during the attempted dislocation of a prosthetic hip, which is proving difficult to achieve. Not only does this technique decrease the risk of an intra-operative distal femoral fracture, but it also aids in the preservation of the already reduced bone stock present during rTHA by reducing the need for trochanteric or acetabular osteotomies.

Our case highlights several factors in which this technique can be useful. The patient presented already had an acetabular protrusion, significant scar tissue formation, as well as pre-operative stiffness. The stage I spacer also introduced a semi-constrained articulation as well as improved soft-tissue tension from leg lengthening. Studies have shown that constrained liners are more difficult to dislocate intraoperatively [4,5]. In order to successfully perform this technique, adequate exposure is necessary, as well as circumferential debridement of all fibrotic tissue about the acetabulum and proximal femur in order to obtain adequate traction. The proximal half of the gluteus maximus tendon is frequently released from its femoral insertion to aid in traction and to decrease some soft tissue tension. It is helpful if one can obtain the patient’s previous implant sizes, as the amount of traction necessary is proportional to the amount of seating of the femoral head onto the trunnion itself regarding plus or minus femoral head sizes. Finally, should this minimalistic technique fail due to difficulty with in-line traction, one can easily proceed with the appropriate osteotomies. 

One of the theoretical complications of this technique is the migration of the femoral head into the pelvis. Per a recent case series and literature review, this complication has been previously reported in 18 cases; however, the rate of this complication was extremely low at the reported institution (0.01%) [13]. Typically, this complication occurs after femoral head dislodgement during a reduction attempt, anterior dislocation resulting in dissociation, or after dissociation during implant trialing. The femoral head component is dissociated from the remainder of the prosthesis, potentially allowing it to migrate along the psoas sheath and into the pelvis. Risk factors for this complication include reduced head-neck ratio, increased impingement, tissue softening, weight loss, increased soft tissue tension, and decreased visualization, which may be the result of obesity (although obesity itself has not been found to be a sole risk factor) [14,15].

Some patients may not require retrieval of the migrated head and function without pain despite sterile trial head retention. A recommended algorithm for the management of such cases does exist; however, most patients described in the previous literature underwent CT with subsequent retrieval by general surgery via an ilioinguinal approach for retroperitoneal components [13]. Some components can be retrieved by extension of the existing incision or another hip approach. Intraperitoneal components must be retrieved via laparotomy. Given that our technique involves in situ dissociation of the head of the femoral component, we feel it is important to be aware of the potential occurrence and manage this complication. Specifically, care should be taken during the retrieval of the dissociated femoral head to prevent unwanted migration into the pelvis.

There are several limitations with respect to our technique. First, it does not directly increase the ability to remove a well-fixed femoral stem from the proximal femur. However, by reducing the need to perform a trochanteric osteotomy and/or attempting to utilize the excessive force required to dislocate the hip, our technique may preserve bone stock and reduce intraoperative fracture incidence, which may help to reduce complications associated with subsequent femoral stem removal. Second, soft tissue tension and fibrotic tissue may still be present and complicate the ability to perform femoral head component-trunnion dislodgement.

## Conclusions

In conclusion, our prosthetic hip dislocation technique during rTHA may be used to overcome intraoperative challenges when standard maneuvers fail. It is also useful in cases where large body habitus, high soft tissue tension, or excessive constraint from components make conventional intraoperative dislocation difficult. The sequential dislocation and retrieval of the components is a useful tool in rTHA that may obviate the need for trochanteric osteotomy, thereby preserving bone stock. It may also help reduce the torsional forces associated with intraoperative dislocation and decrease the risk of periprosthetic fracture. The senior author has used this technique in over 20 rTHA cases in the settings of morbid obesity, heterotopic ossification, hemiarthroplasty to total hip arthroplasty conversions, components that protruded medially, dislocation of dual mobility constructs, and dislocation of constrained constructs. To the best of our knowledge, this technique has not been described previously in the literature.
